# Predicting the quality of soybean seeds stored in different environments and packaging using machine learning

**DOI:** 10.1038/s41598-022-12863-5

**Published:** 2022-05-25

**Authors:** Geovane da Silva André, Paulo Carteri Coradi, Larissa Pereira Ribeiro Teodoro, Paulo Eduardo Teodoro

**Affiliations:** 1 Department of Agronomy, Campus de Chapadão do Sul, Federal University of Mato Grosso do Sul, Chapadão do Sul, MS 79560-000 Brazil; 2 Department Agricultural Engineering, Rural Sciences Center, Federal University of Santa Maria, Avenue Roraima, 1000, Camobi, Santa Maria, Rio Grande do Sul 97105-900 Brazil; 3 Department of Agricultural Engineering, Laboratory of Postharvest, Campus Cachoeira do Sul, Federal University of Santa Maria, Highway Taufik Germano, 3013, Passo D’Areia, Cachoeira do Sul, Rio Grande do Sul 96506-322 Brazil

**Keywords:** Environmental sciences, Engineering, Mathematics and computing

## Abstract

The monitoring and evaluating the physical and physiological quality of seeds throughout storage requires technical and financial resources and is subject to sampling and laboratory errors. Therefore, machine learning (ML) techniques could help optimize the processes and obtain accurate results for decision-making in the seed storage process. This study aimed to analyze the performance of ML algorithms from variables monitored during seed conditioning (temperature and packaging) and storage time to predict the physical and physiological quality of stored soybean seeds. Data analysis was performed using the Artificial Neural Networks, decision tree algorithms REPTree and M5P, Random Forest, and Linear Regression. In predicting seed quality, the combination of the input variables temperature and storage time for REPTree and Random Forest algorithms outperformed the linear regression, providing higher accuracy indices. Among the most important results, it was observed for apparent specific mass that T + P + ST, T + ST, P + ST, and ST had the highest r means and the lowest MAE means, however, Person's r coefficient for these inputs was 0.63 and the MAE between 9.59 to 10.47. The germination results for inputs T + P + ST and T + ST had the best results (r = 0.65 and r = 0.67, respectively) in the ANN, REPTree, M5P and RF models. Using computational intelligence algorithms is an excellent alternative to predict the quality of soybean seeds from the information of easy-to-measure variables.

## Introduction

In post-harvest, the storage stage is intended to preserve the quality of the seeds^1,2^. However, variations in seed moisture content, shape, environment, and storage time can influence the metabolic activity and physiological quality of seeds^3,4^. The increase in the respiratory rate of the grain mass cause continuous transformations in the grains, since organic matter, when in contact with oxygen, is transformed into CO_2_ and H_2_O, releasing energy in the form of heat, resulting in a more favorable environment for the infestation of insect pests, mites, fungal infection, physical–chemical and physiological variations^4,5^.

To reduce the metabolic activity of the seeds, it is suggested to control the temperature and relative humidity of the storage environment so that the seeds remain in hygroscopic equilibrium with moisture contents close to 12% (w.b.), which is considered a safe storage humidity^5–7^. For this, artificial cooling technology has been used^8,9^. Maintaining the seeds at low temperatures, associated with a controlled condition of relative humidity, can provide a favorable storage condition. Reducing the temperature on the grain mass can reduce the speed of biochemical and metabolic reactions of the grains where the reserves in the support tissue are unfolded, transported and resynthesized in the embryonic axis, allowing the maintenance of the initial characteristics of the stored grains for longer periods^6–9^. Besides this, the use of hermetic or semi-hermetic packaging can contribute to the reduction of seed respiration and maintenance of quality^10–13^.

In order to obtain more accurate information about the quality of stored seeds, especially regarding the apparent specific mass and germination as a function of storage conditions and time, the application of predictive computer algorithms is recommended. In this context, the use of Machine Learning (ML) algorithms can provide improve data processing and analysis ability. When adequately modeled, ML techniques can provide answers in a shorter time when compared to statistical regression models.

In recent years, ML methods have been used to predict crop yield^14,15^, application rate of nitrogen to soils^16^ and leaf nitrogen concentration^17^, classify seeds^18^, reduce phosphorus in wastewater^19^, and reduce crude protein in stored grain^20^. Random Forest algorithm, for example, is an ML technique used successfully in crop prediction^21^. Compared to multiple linear regression models, this technique is effective and easier to use in yield prediction analyses for maize^15^, soybean^14^, and potatoes^22^. Another example is Artificial Neural Networks (ANNs), which are algorithms that can be trained^23,24^ to analyze and interpret complex food safety data, physical and chemical predictions^23,25^.

To fill gaps where conventional statistics cannot generate satisfactory prediction results, data modeling using ML techniques may become a viable alternative for evaluating the quality of stored soybean seeds instead of conducting time-consuming and costly tests in laboratories. In seed processing and storage units, the use of ML can be an auxiliary tool for decision-making within the seed storage environment, thereby contributing to process optimization and loss reduction, impacting socio-economically in the production environment and collaborating for the formation of a more sustainable post-harvest system. Thus, this study aimed to analyze the performance of ML algorithms from soybean seed conditioning variables (temperature, packaging) and storage time to predict physical and physiological quality of stored soybean seeds.

## Material and methods

### Characterization of the experiments

**Figure 1:**
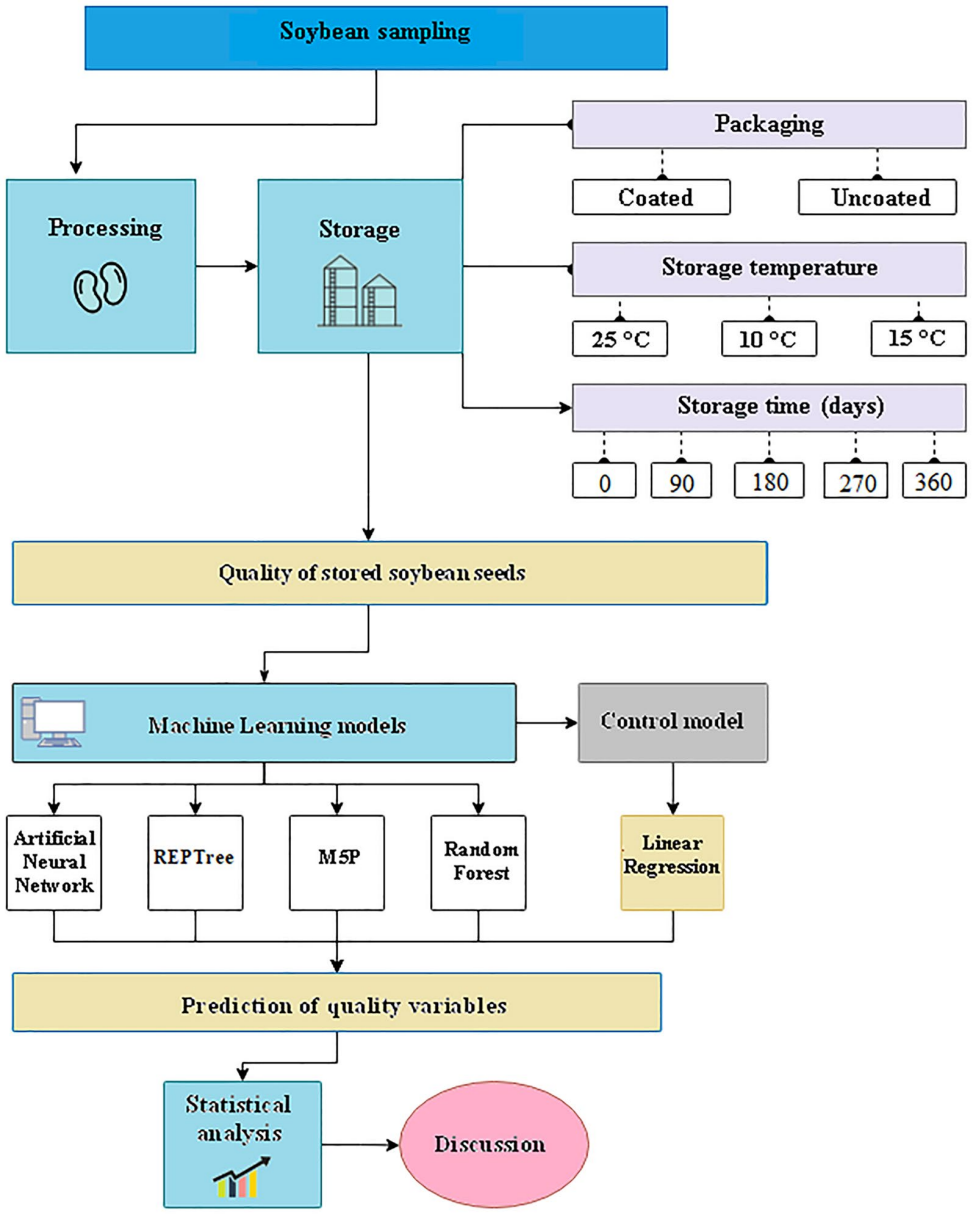
Experimental scheme.

A completely randomized was used, three-factor (3 × 2 × 5) experiment experimental design: three storage temperatures (10, 15, and 25 °C), two packagings (raffia bag and polyethylene coated raffia bag), and five evaluation times (0, 3, 6, 9, and 12 months). Every three months, three packagings (i.e., three repetitions) of each treatment were sampled to make quality assessments. After this procedure, the packaging was discarded. Figure 1 represents the experimental setup.

The raffia bags were made of 20 cm (wide) × 30 cm (height) × 0.25 cm polypropylene material. The polyethylene coating used to store the grains in the raffia bags had dimensions of 20 cm (wide) × 30 cm (height) × 0.1 cm (thick of high density) being produced by the company specialized in food packaging (Videplast Company, Videira, Santa Catarina, Brazil).

The polyethylene packages were constituted by partially crystalline and flexible thermoplastic resin material obtained through the ethylene polymerization, having low density, high tenacity, good impact resistance, flexibility, easy processability, electrical properties and stability, and low permeability to water. It is formed by polar organic compounds and can be changed by the temperature environment. To assess the effects of the storage environments on the physical quality of the soybean grains, the three conditions (packaging, temperatures conditions, and storage time) were grouped to define the storage environments (Table 1).

**Table 1:** Experimental design and grouping of storage environments.

Packaging	Storage temperature (°C)	Storage time (months)
With coating	25	0
With coating	25	3
With coating	25	6
With coating	25	9
With coating	25	12
With coating	15	0
With coating	15	3
With coating	15	6
With coating	15	9
With coating	15	12
With coating	10	0
With coating	10	3
With coating	10	6
With coating	10	9
With coating	10	12
Uncoating	25	0
Uncoating	25	3
Uncoating	25	6
Uncoating	25	9
Uncoating	25	12
Uncoating	15	0
Uncoating	15	3
Uncoating	15	6
Uncoating	15	9
Uncoating	15	12
Uncoating	10	0
Uncoating	10	3
Uncoating	10	6
Uncoating	10	9
Uncoating	10	12

### Sampling and quality analysis of soybean seeds

The soybean grains were obtained from the production fields of a rural property in the municipality of Chapadão do Céu-GO, Brazil, and were cleaned to remove impurities and foreign matter LC 160 machine (Kepler Weber, Panambi, Rio Grande do Sul, Brazil). Then, they were dried in drying silos with radial airflow (Rome Silos Company, Cambé, Paraná, Brazil). The dryer is built in modulated wooden panels (2.11 m × 0.60 m) with treated boards interspersed with aluminum shutters, fixed by galvanized wire and structured with laminated angle arches, mounted overlapping on a self-draining metallic background. Radial ventilation through central tube and centrifugal fan. The temperature of the grain drying air, up to 12% (w.b.) of moisture content, was 40 °C. Then, the grains were processed using spiral separator equipment (Akyurek Technology, Mersin, Turkey) and a dissymmetric table model SDS-80 (Silomax, Rolândia, Paraná, Brazil) in order to standardize their size and weight. The grains lots were stored in raffia bags (polypropylene) in air-conditioned warehouses with temperature control. Nine-kilogram grain samples were collected from the bags using a sampler (EAGRI Equipments, Panambi, Rio Grande do Sul, Brazil), in, with the aid of a manual presser order to be stored experimentally in different storage environments.

During the storage period, the temperature of the grain mass was monitored weekly with the aid of a digital thermohygrometer model Logbox-RHT-LCD (Novus Electronic Products Company, Canoas, Rio Grande do Sul, Brazil) and every three months, the grain samples were collected for quality assessment. The moisture content of the grains was determined in a forced air circulation oven at 220 L (Tecnal Company, Piracicaba, São Paulo, Brazil) at 105 °C ± 1 °C, for 24 h, with four repetitions. Then, the samples were removed and placed in a desiccator for cooling at 5 L (Tecnal Company, Piracicaba, São Paulo, Brazil) and subsequent weighing at balance model B13200H (Shimadzu, São Paulo, Brazil) according to the recommendations of the Rule for Seed Analysis^26^. The moisture content was determined by the mass difference of the initial and the final sample, and the results were expressed as a percentage (w.b.). The apparent specific mass of the grains was determined with the aid of a 150 mL beaker and a precision scale, using the mass/volume ratio, with four repetitions^26^.

The electrical conductivity evaluation was carried out with four sub-samples, each containing 25 seeds per experimental unit, weighed on a precision scale of 0.001 g, and placed in plastic cups with 75 mL of distilled water, and was undertaken in a incubator at 25 °C, for 24 h. After imbibition, the electrical conductivity of the immersion solution was obtained with the aid of a digital conductivity meter model CD-21 (Digimed, São Paulo, Brazil) and the results were expressed in μS cm^−1^ g^−1^ according to the methodology proposed by Brazil^26^. For the vigor and germination tests, four sub-samples of 50 seeds from each experimental unit were used, distributed in paper towel rolls (Germitest), and moistened with distilled water in an amount that was 2.5 times the dry paper mass. Then, the rolls with the seeds were placed in a germinator model Mangesdorf (Tecnal, Piracicaba, São Paulo, Brazil) set at a temperature of 25 °C ± 2 °C. The evaluations were carried out on the fifth (vigor) and eighth (germination) days after the test was installed, by counting normal and abnormal seedlings as well as dead seeds, according to the criteria established in the Rules for Seed Analysis^26^.

### Machine learning models

The models tested were: Artificial Neural Network (ANN), decision tree algorithms REPTree and M5P, Random Forest (RF), and Linear Regression (LR). The ANN tested consists of a single hidden layer formed by a number of neurons that is equal to the number of attributes plus the number of classes, all divided by 2^27^. REPTree model is an adaptation of the C4.5 classifier that can be used in regression problems with an additional pruning step based on an error reduction strategy^28^. M5P model is a reconstruction of Quinlan's M5 algorithm based on the conventional decision tree with the addition of a linear regression function to the leaf nodes^29^. RF model can produce several prediction trees for the same data set and use a voting scheme among all learned trees to predict new values^30^. RL model was used as a control model as it serves to predict the behaviors between variables that have a good correlation, and is a widely used model in statistics.

The prediction of the variables moisture content (MC), apparent specific mass (ASM), electrical conductivity (EC), germination (G), and vigor (V) in soybean seeds was performed by all machine learning (ML) models in a tenfold stratified randomized cross-validation with 10 repetitions (100 runs for each model). Different inputs were considered for each model in predicting these variables: temperature (T), packaging (P), storage time (ST), T + P, T + ST, T + P + ST.

The statistics used to verify the quality of fit were Pearson's correlation coefficient (r) between the observed and predicted values by each model and the mean absolute error (MAE) of the predicted values in relation to the observed ones. ML analyses were performed with Weka 3.9.4 software using the default configuration for all models tested^31^ on an Intel® CoreTM i5 CPU with 4 Gb of RAM.

### Statistical analysis

After obtaining the correlation coefficient (r) and the mean absolute error (MAE) statistics, an analysis of variance considering a two factorial scheme (models versus inputs) with 10 repetitions (folds) was performed. The r varies between 0 and 1, and its proximity to 1 indicates that the model is better at explaining the variability of the sample data. It is expected an MAE result inverse to those of the correlation coefficient since it is used to analyze the error between the values predicted by the model and those expected; the lower the values, the closer the model is to the observed outputs. The means were grouped by the Scott-Knott test at 5% probability. Bar charts were constructed for each variable (r and MAE) considering the models and inputs tested. These analyses were performed on the R software^32^ using the ExpDes.pt and ggplot2 packages.

### Ethics declarations

The experimental research and field studies on plants and plant material were comply with local and national regulations. The authors had permission to collect grains, attending local, national, and international regulations.The study complied with institutional, national, and international guidelines and legislation.

## Results and discussion

### Analysis of variance

Table 2 shows the p-value results (r and MAE) for the prediction of the variables evaluated, considering the different ML models (M) and different inputs (I). It was possible to observe that there was significant interaction (p < 0.05) between factors for r and MAE for the variables moisture content and germination, and MAE for electrical conductivity. The r of the apparent specific mass had a significant effect only for the inputs, while for MAE there was significant variation for M and I. MAE of the variable ASM and the r of the variables EC and V had significant variation for M and I.

**Table 2:** The P-value from the analysis of variance for Pearson's correlation coefficient (r) between observed and estimated values of moisture content (MC), apparent specific mass (ASM), electrical conductivity (EC), germination (G), and vigor (V) of soybean seeds by different machine learning models and inputs.

Sources of variation	MC		ASM		EC		G		V	
	r	MAE	r	MAE	R	MAE	r	MAE	r	MAE
Models (M)	< 0.00	< 0.00	0.99	0.00	0.03	< 0.00	< 0.00	< 0.00	0.02	0.00
Inputs (I)	< 0.00	< 0.00	0.00	0.00	0.00	< 0.00	< 0.00	< 0.00	0.00	0.00
MxI	< 0.00	< 0.00	1.00	1.00	0.43	< 0.00	< 0.00	< 0.00	0.40	0.64

### Moisture content

During storage, biological processes in the products continue to occur with greater or lesser intensity, depending on storage conditions and the moisture content of the products^33^. Thus, it was observed that the inputs T + P + ST and the combination T + ST were the ones that had the best performance in predicting seed quality. Juvino et al.^34^ observed a higher range of moisture content in uncontrolled temperature environments than the acclimatized one at 18 °C. When the seeds were subjected to lower storage temperatures, they remained in hygroscopic equilibrium with moisture contents close to the initial storage conditions^35^.

The reduction in grain temperature slows down the biochemical and metabolic reactions of the seeds, which reserves stored in the support tissue are unfolded, transported and resynthesized in the embryonic axis and allow the maintenance of the initial characteristics of seed storage for longer periods. The combination of input variables temperature and storage time was the best moisture content predictor of soybean seed indices during the storage period. The moisture content of soybean seeds for safe storage is 12% (w.b.), which must remain in equilibrium moisture content with intergranular air at 65–67%^35^. The prediction of seed moisture content during storage is of paramount importance, since the increase or reduction of moisture content can influence the metabolic activity of the seeds, in the cellular tissues and, consequently, in the physiological quality.

For inputs T, T + P, P, P + ST, and ST, there was no difference between the models tested (Tables 3 and 4). However, for inputs T + P + ST and T + ST, the ANN, REPTree, M5P, and RF models had the highest means compared to LR. When analyzing the inputs within each model, it can be seen that, regardless of the model, the T + P + ST configuration provided the highest r means. The MAE results for the ML algorithms with T + ST + P and T + ST as inputs ranged from 0.30 to 0.41, while LR scored 0.73. For the T + P + ST configuration, all ML models had r values above or equal to 0.94, while for the LR the observed r was 0.72.

**Table 3:** Unfolding the significant interaction between model x input for Pearson's correlation coefficient (r) between the observed and estimated values of moisture content in soybean seeds by different machine learning models and inputs.

Models	T	P + T	ST + P + T	ST + T	P	ST + P	ST
ANN	0.36 aE	0.43 aD	0.94 aA	0.86 aB	0.10 aF	0.63 aC	0.63 aC
REPTree	0.36 aE	0.43 aD	0.95 aA	0.87 aB	0.10 aF	0.63 aC	0.63 aC
LR	0.36 aC	0.37 aC	0.72 bA	0.72 bA	0.10 aD	0.63 aB	0.63 aB
M5P	0.36 aE	0.43 aD	0.94 aA	0.87 aB	0.10 aF	0.63 aC	0.63 aC
RF	0.36 aE	0.43 aD	0.95 aA	0.87 aB	0.10 aF	0.63 aC	0.63 aC

Means followed by equal lowercase letters in the same column and equal uppercase letters in the same row do not differ by the Scott-Knott test at 5% probability.

*T* temperature, *P* packaging, *ST* storage time.

**Table 4:** Unfolding the significant interaction between model x input for mean absolute error (MAE) between the observed and estimated values of moisture content in soybean seeds by different machine learning models and inputs.

Models	T	P + T	ST + P + T	ST + T	P	ST + P	ST
ANN	1.26 aA	1.22 aA	0.41 bD	0.67 aC	1.33 aA	0.92 aB	0.92 aB
REPTree	1.07 bA	1.09 bA	0.30 bD	0.53 bC	1.11 bA	0.80 aB	0.81 aB
LR	1.07 bA	1.09 bA	0.73 aB	0.73 aB	1.11 bA	0.81 aB	0.81 aB
M5P	1.07 bA	1.09 bA	0.32 bC	0.53 bD	1.11 bA	0.81 aB	0.81 aB
RF	1.07 bA	1.09 bA	0.30 bC	0.53 bD	1.11 bA	0.81 aB	0.81 aB

Means followed by equal lowercase letters in the same column and equal uppercase letters in the same row do not differ by the Scott-Knott test at 5% probability.

*T* temperature, *P* packaging, *ST* storage time.

**Figure 2:**
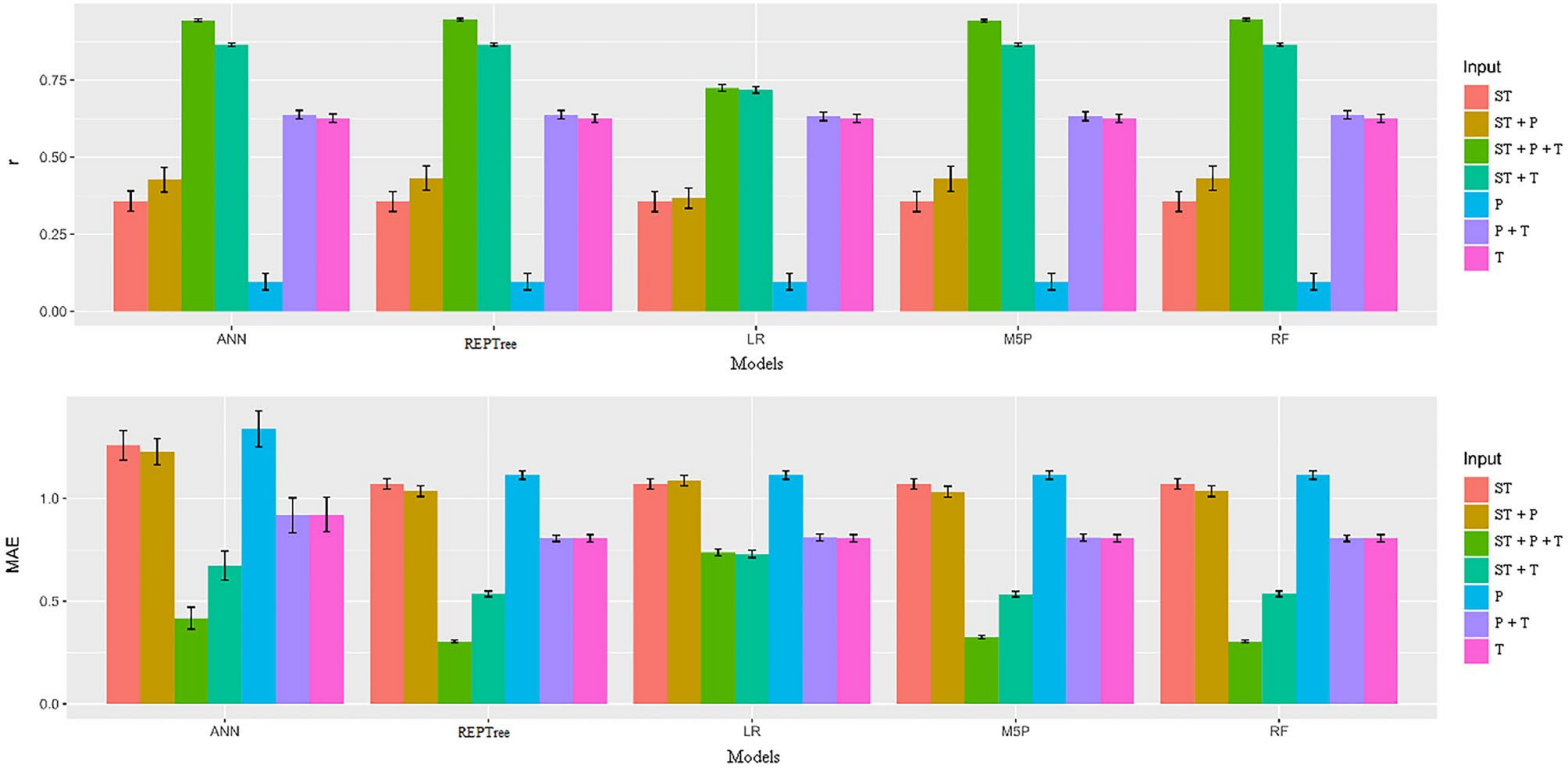
Mean values and scatter plot for the Pearson's correlation coefficient (r) and mean absolute error (MAE) between observed and estimated values of moisture content in soybean seeds by different machine learning models and inputs.

Inputs T, T + P, and P from the ANN model had the highest means (Table 4), while for input T + P + ST the LR model had the highest mean. For the T + ST input, the ANN and LR models showed the highest means, while for the P + ST and ST inputs there were no statistical differences among the models tested. It is important to highlight that MAE behaved contrary to r. The low MAE values represented a higher proximity between the observed and estimated values. When analyzing the inputs within each model, it was possible to observe that the T + P + ST configuration provided the lowest MAE means regardless of the model. In Fig. 2, it was observed that the ANN, REPTree, M5P, and RF models when associated with inputs T + P + ST and T + ST provided the highest r and lowest MAE values. Therefore, Random Forest algorithm is recommended to predict the moisture content of the seeds during the storage period because used a smaller amount of data, making it possible to better conduct overfitting problems.

### Apparent specific mass

The ASM did not differ for r in the tested models. However, the ANN model presented the highest average MAE in relation to the others, which indicates that this model overestimated the apparent specific mass values. Regarding the inputs tested, it was possible to observe in Tables 5 and 6 that T + P + ST, T + ST, P + ST, and ST showed the highest r means and the lowest MAE means. Person's r coefficient for these inputs was 0.63 and the MAE between 9.59 to 10.47.

**Table 5:** Clustering of means for the Pearson's correlation coefficient (r) and mean absolute error (MAE) between observed and estimated values of apparent specific mass in soybean seeds by different learning models.

Models	r	MAE
ANN	0.35 a	17.12 a
REPTree	0.35 a	10.45 b
LR	0.35 a	10.45 b
M5P	0.36 a	10.45 b
RF	0.35 a	10.44 b

Means followed by the same letters in the same column do not differ by the Scott-Knott test at 5% probability.

**Table 6:** Clustering of means for the Pearson's correlation coefficient (r) and mean absolute error (MAE) between observed and estimated values of apparent specific mass in soybean seeds by different inputs.

Input	r	MAE
T	0.00 b	13.86 a
P + T	− 0.02 b	13.88 a
ST + P + T	0.63 a	10.47 b
ST + T	0.63 a	10.31b
P	− 0.02 b	13.88 a
ST + P	0.63 a	10.47 b
ST	0.63 a	9.59 b

Means followed by the same letters in the same column do not differ by the Scott-Knott test at 5% probability.

*T* temperature, *P* packaging, *ST* storage time.

In the ASM prediction, storage time was the condition present in all input combinations that best predicted the variable levels. A study carried out by Alencar et al.^36^ verified that the ASM was changed according to temperature and storage time conditions. According to the findings reported by the Alencar et al^36^, the decrease in apparent specific mass occurred after 180 days of storage due to the increased metabolic activity of the grains influenced by variations in moisture content and temperature of the stored seed mass.

**Figure 3:**
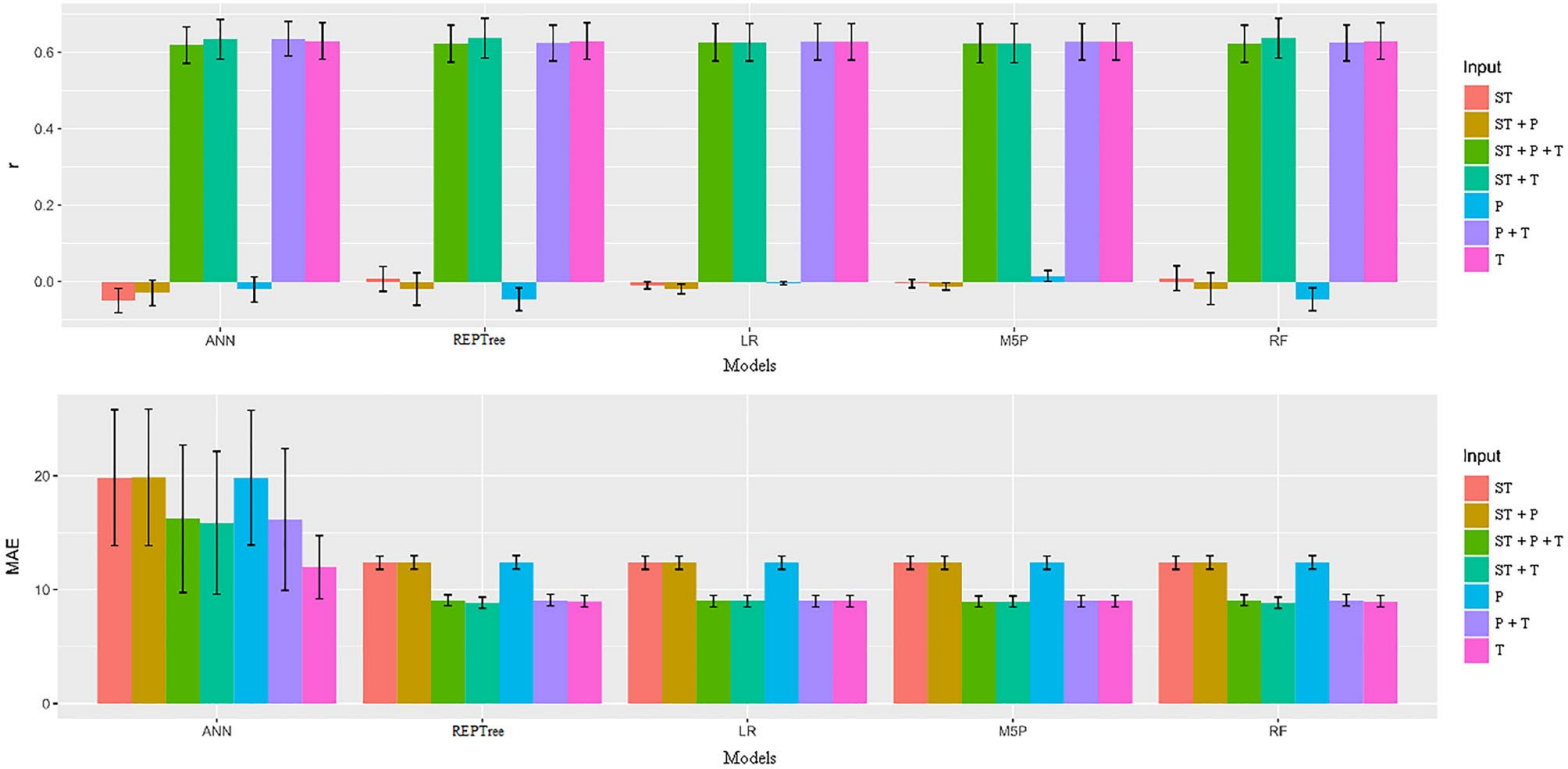
Mean values and scatter plot for the variables Pearson's correlation coefficient (r) and mean absolute error (MAE) between observed and estimated values of apparent specific mass in soybean seeds by different machine learning models and inputs.

Figure 3 shows that the REPTree, M5P, and RF models when associated with inputs T + P + ST, T + ST, P + ST and ST provided the highest r values and lowest MAE values. Importantly, while the ANN model had the best r results with the aforementioned inputs, this model also had high ANN values (17.12) for all inputs. Furthermore, no model had statistically different results from the LR model.

The results obtained indicated that the storage time had a greater influence in the ASM, that is, it reduced the seed mass in relation to their volume. This loss occurs due to the chemical reactions of oxidation during the respiratory process of the seeds, which consume accumulated energy in the form of organic compounds such as sugars, starches and others, effectively reducing the mass and, therefore, the weight of the seeds^5,8,9^. This result indicates that the seeds suffered deterioration and losses in physiological quality. The ANN model can be used to predict the ASN variation.

### Electrical conductivity

No significant difference was observed for EC considering the models analyzed (Table 7). However, even so, the r value for the LR model was lower when compared to the other models tested. Regarding the different inputs for EC (Table 8), the combination T + P + ST and T + ST had the highest r means (0.65 and 0.63, respectively), while the input T had the lowest r mean. For inputs T, T + P, P, P + ST and ST, the MAE values did not differ among the models tested. The lowest means were verified for inputs T + P + ST and T + ST for the models REPTree, M5P, and RF (Table 9).

**Table 7:** Clustering of means for the Pearson's correlation coefficient (r) and mean absolute error (MAE) between observed and estimated values of electrical conductivity in soybean seeds by different learning models.

Models	r
ANN	0.41 a
REPTree	0.42 a
LR	0.38 b
M5P	0.42 a
RF	0.42 a

Means followed by the same letters in the same column do not differ by the Scott-Knott test at 5% probability.

**Table 8:** Clustering of means for the Pearson's correlation coefficient (r) between observed and estimated values of electrical conductivity in soybean seeds by different inputs.

Input	r
T	0.32 c
P + T	0.34 c
ST + P + T	0.65 a
ST + T	0.63 a
P	0.03 d
ST + P	0.45 b
ST	0.44 b

Means followed by the same letters in the same column do not differ by the Scott-Knott test at 5% probability.

*T* temperature, *P* packaging, *ST* storage time.

**Table 9:** Unfolding the significant interaction between model x input for mean absolute error (MAE) between the observed and estimated values of electrical conductivity in soybean seeds by different machine learning models and inputs.

Models	T	P + T	ST + P + T	ST + T	P	ST + P	ST
ANN	29.91 aA	30.43 aA	25.13 aB	25.24 aB	31.12 aA	30.23 aA	30.35 aA
REPTree	28.25 aA	28.33 aA	21.67 bB	21.94 bB	29.93 aA	26.61 aA	26.80 aA
LR	28.25 aA	28.37 aA	25.47 aB	25.39 aB	29.95 aA	26.81 aB	26.77 aB
M5P	28.25 aA	1.03 bC	21.60 bB	22.01 bB	29.95 aA	26.78 aA	26.77 aA
RF	28.26 aA	28.34 aA	21.67 bB	21.95 bB	29.94 aA	26.61 aA	26.81 aA

Means followed by equal lowercase letters in the same column and equal uppercase letters in the same row do not differ by the Scott-Knott test at 5% probability.

*T* temperature, *P* packaging, *ST* storage time.

Considering that conditions (packaging, temperature, and relative humidity) and storage time can influence seed moisture contents by causing seed drying or rewetting, it is expected that the prediction of electrical conductivity as a function of the input conditions tested indicates deterioration of cellular tissues and seed quality. Alencar et al.^36^, when evaluating the soybean quality by the electrical conductivity test, observed that the interaction between moisture content, temperature, and storage time were significant and influenced the quality of the seeds. Carvalho et al.^37^ and Coradi et al.^38^ observed that the most significant increase in conductivity of soybean seeds occurred after 180 days of storage, indicating changes in the cellular tissues of the seeds.

**Figure 4:**
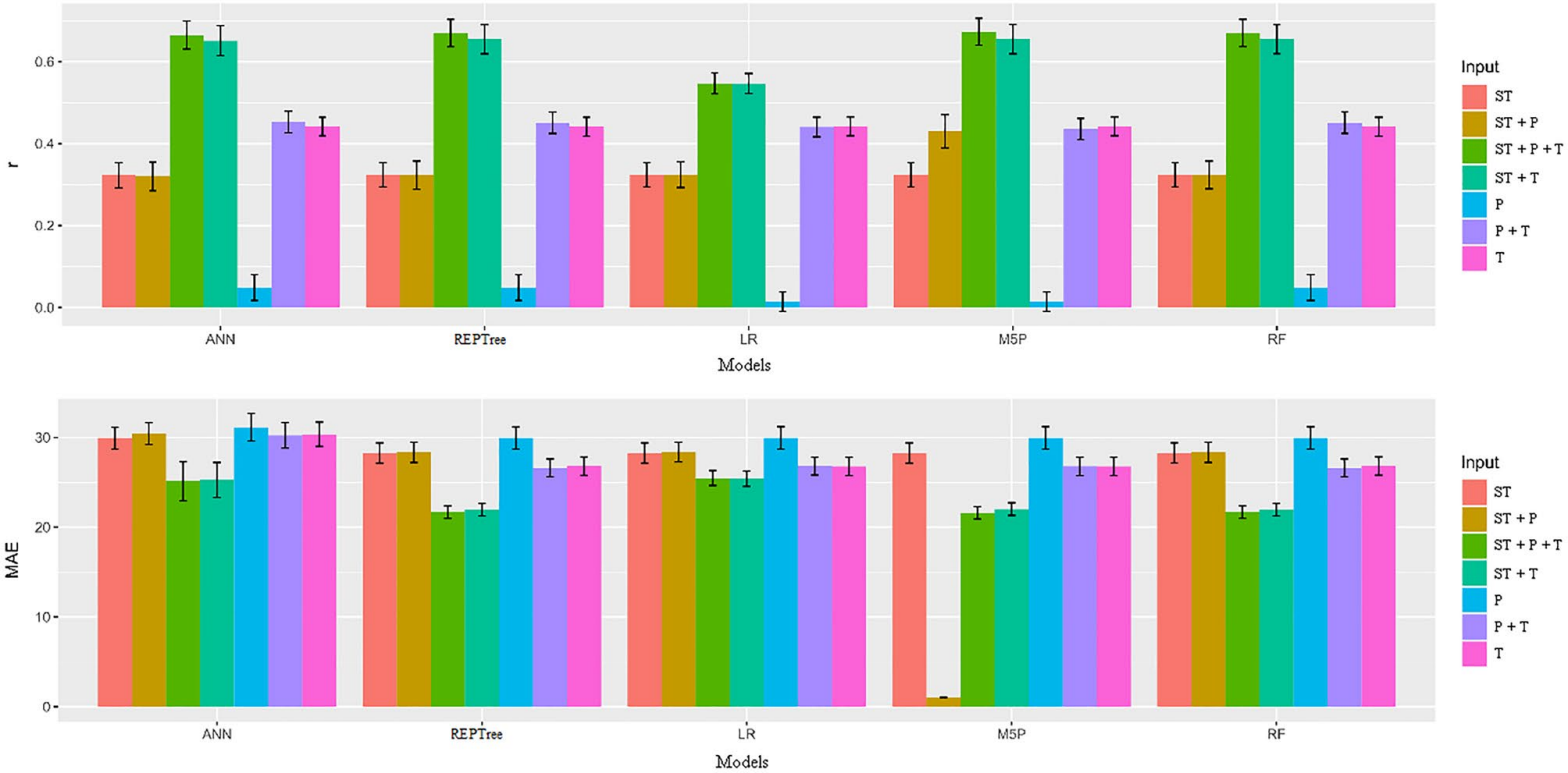
Mean values and scatter plot for the variables Pearson's correlation coefficient (r) and mean absolute error (MAE) between observed and estimated values of electrical conductivity in soybean seeds by different machine learning models and inputs.

In Fig. 4, it can be seen that the T + P + ST inputs obtained the best MAE results (21.67) for REPTree, M5P, and RF models. Similar results were observed for the T + ST inputs, where the MAE ranged from 21.95 to 22.01. Although the ANN model showed satisfactory r results, the MAE values did not differ from the LR model.

Therfore, the effect of temperature associated with storage time had a greater influence on the deterioration of cell membranes determined by the electrical conductivity test. Random Forest was the algorithm that better predicted electrical conductivity results, for the same reasons described for the variable water contents, smaller amount of data, making it possible to better conduct overfitting problems.

### Germination

The obtained and estimated values for soybean seed germination are presented in Tables 10 and 11. The inputs T, T + P, P, and ST did not show significant variation. The highest means for inputs T + P + ST and T + ST were obtained in the ANN, REPTree, M5P, and RF models, while for the REPTree model the best results were obtained at input P + ST.

**Table 10:** Unfolding the significant interaction between model x input for Pearson's correlation coefficient (r) between the observed and estimated values of germination in soybean seeds by different machine learning models and inputs.

Models	T	P + T	ST + P + T	ST + T	P	ST + P	ST
ANN	0.33 aB	0.33 Ab	0.67 aA	0.65 aA	0.03 aC	0.36 bB	0.35 aB
REPTree	0.33 aB	0.33 aB	0.67 aA	0.65 aA	0.03 aC	0.65 aA	0.35 aB
LR	0.33 aB	0.33 aB	0.48 bA	0.48 bA	-0.02 aC	0.35 bB	0.35 aB
M5P	0.33 aB	0.32 aB	0.66 aA	0.65 aA	-0.02 aC	0.36 bB	0.35 aB
RF	0.33 aB	0.33 aB	0.67 aA	0.65 aA	0.03 aC	0.36 bB	0.35 aB

Means followed by equal lowercase letters in the same column and equal uppercase letters in the same row do not differ by the Scott-Knott test at 5% probability.

*T* temperature, *P* packaging, *ST* storage time.

**Table 11:** Unfolding the significant interaction between model x input for mean absolute error (MAE) between the observed and estimated values of germination in soybean seeds by different machine learning models and inputs.

Models	T	P + T	ST + P + T	ST + T	P	ST + P	ST
ANN	13.33 Aa	13.65 bA	9.77 aC	11.61B	13.70 aA	14.71 aA	12.16 aB
REPTree	11.75 aA	11.76 bA	8.95 aB	9.10 bB	12.67 aA	9.10 cB	11.89 aA
LR	11.77 aA	11.79 bA	11.26 aA	11.25 aA	12.67 aA	11.89 bA	11.87 aA
M5P	11.77 aA	11.84 bA	9.05 aB	9.21 bB	12.67 aA	11.89 bA	11.87 aA
RF	11.76 aB	17.01 aA	8.95 aC	9.10 bC	12.68aB	11.92 bB	11.89 aB

Means followed by equal lowercase letters in the same column and equal uppercase letters in the same row do not differ by the Scott-Knott test at 5% probability.

*T* temperature, *P* packaging, *ST* storage time.

**Figure 5:**
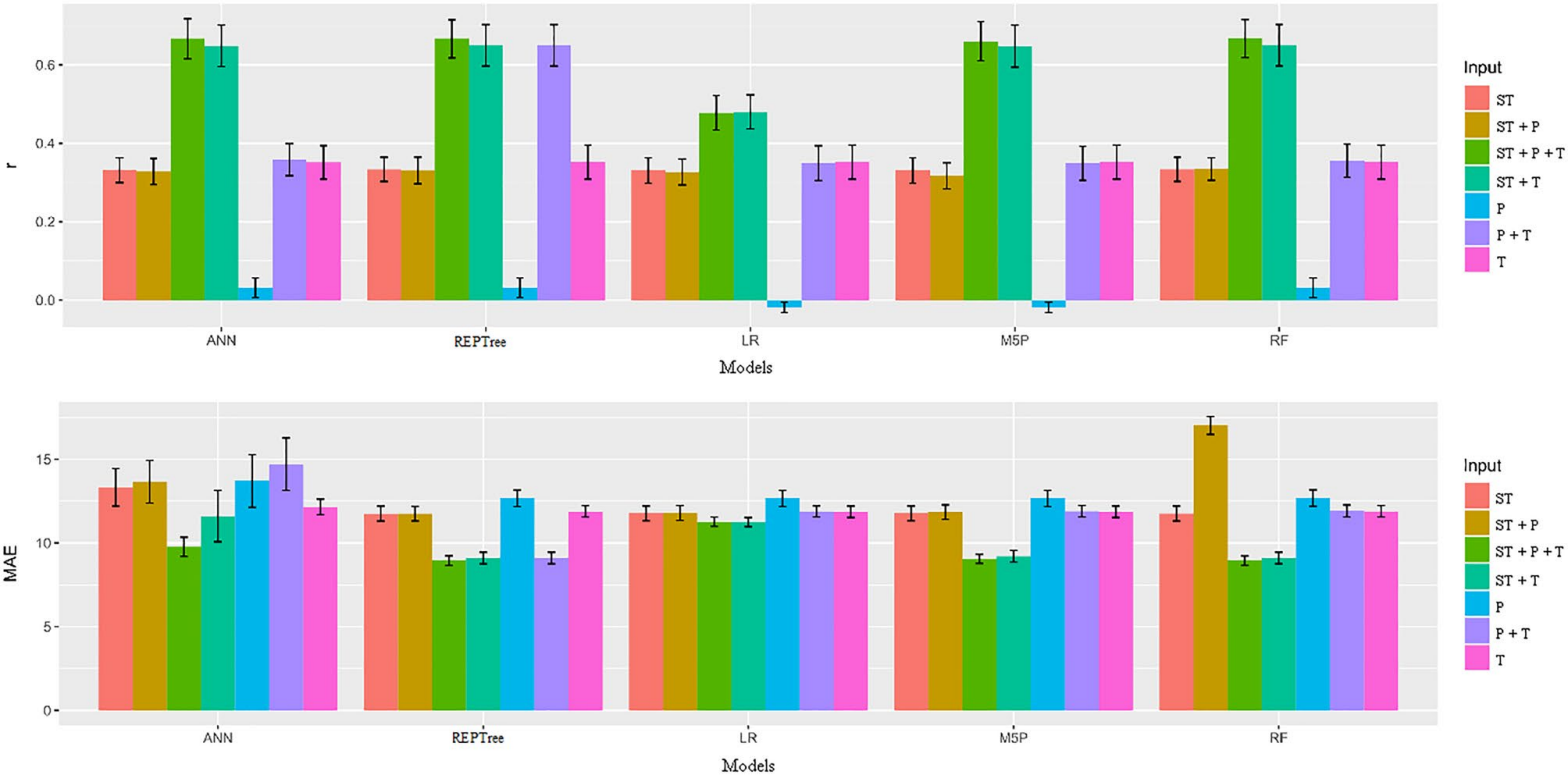
Mean values and scatter plot for the variables Pearson's correlation coefficient (r) and mean absolute error (MAE) between observed and estimated values of germination in soybean seeds by different machine learning models and inputs.

In Table 10 are the unfoldings of the significant interactions between the models and inputs, considering the observed and estimated seed germination values for MAE. The LR model obtained the highest MAE value (11.26) for the input combination T + P + ST. The REPTree and RF models had the lowest MAE (8.95) for the T + P + ST and T + ST combination. For inputs T, T + P, P, and ST, the means were higher and input P + ST, where only the REPTree model showed a low mean (Fig. 5).

High percentages of seed germination are obtained over storage time when seeds are stored in proper temperatures and packaging^39^. Coradi et al.^40^ verified that the artificially cooled soybean seeds maintained their physiological quality for 140 days of storage. Coradi et al.^41^ observed that seeds stored in uncontrolled environments obtained increased respiration rate and accelerated deterioration. It was found in Table 11 that the germination results for inputs T + P + ST and T + ST had the best results (r = 0.65 and r = 0.67, respectively) in the ANN, REPTree, M5P and RF models. The LR model had a low performance (r = 0.48) for the inputs T + P + ST and T + ST, as did the ANN model for the input T + ST.

The germination results followed the results obtained with the moisture contents, apparent specific mass and electrical conductivity. However, in addition to the temperature and storage time factors, the relationship between storage time and packaging had a very significant influence on the physiological quality of the seeds. Among the models tested, REPTree model stood out among the others.

### Vigor

The statistics obtained for the vigor variable (r and MAE) showed no significant interaction between models and inputs. However, the ANN model presented the highest mean MAE in relation to the others (Table 12), indicating that the ANN model overestimated the vigor values. Regarding the inputs tested, it was possible to observe (Table 13) that T + P + ST, T + ST, P + ST, and ST presented the highest mean r and the lowest mean MAE.

**Table 12:** Clustering of means for the Pearson's correlation coefficient (r) and mean absolute error (MAE) between observed and estimated values of vigor in soybean seeds by different learning models.

Models	r	MAE
ANN	0.44 a	17.33 a
REPTree	0.44 a	15.46 c
LR	0.39 b	16.20 b
M5P	0.44 a	15.51 c
RF	0.43 a	15.46 c

Means followed by the same letters in the same column do not differ by the Scott-Knott test at 5% probability.

**Table 13:** Clustering of means for the Pearson's correlation coefficient (r) and mean absolute error (MAE) between observed and estimated values of vigor in soybean seeds by different inputs.

Input	r	MAE
T	0.34 c	17.23 b
P + T	0.33 c	17.27 b
ST + P + T	0.68 a	13.09 d
ST + T	0.68 a	13.24 d
P	0.01 d	18.56 a
ST + P	0.47 a	16.29 c
ST	0.47 a	26.28 c

Means followed by the same letters in the same column do not differ by the Scott-Knott test at 5% probability.

*T* temperature, *P* packaging, *ST* storage time.

**Figure 6:**
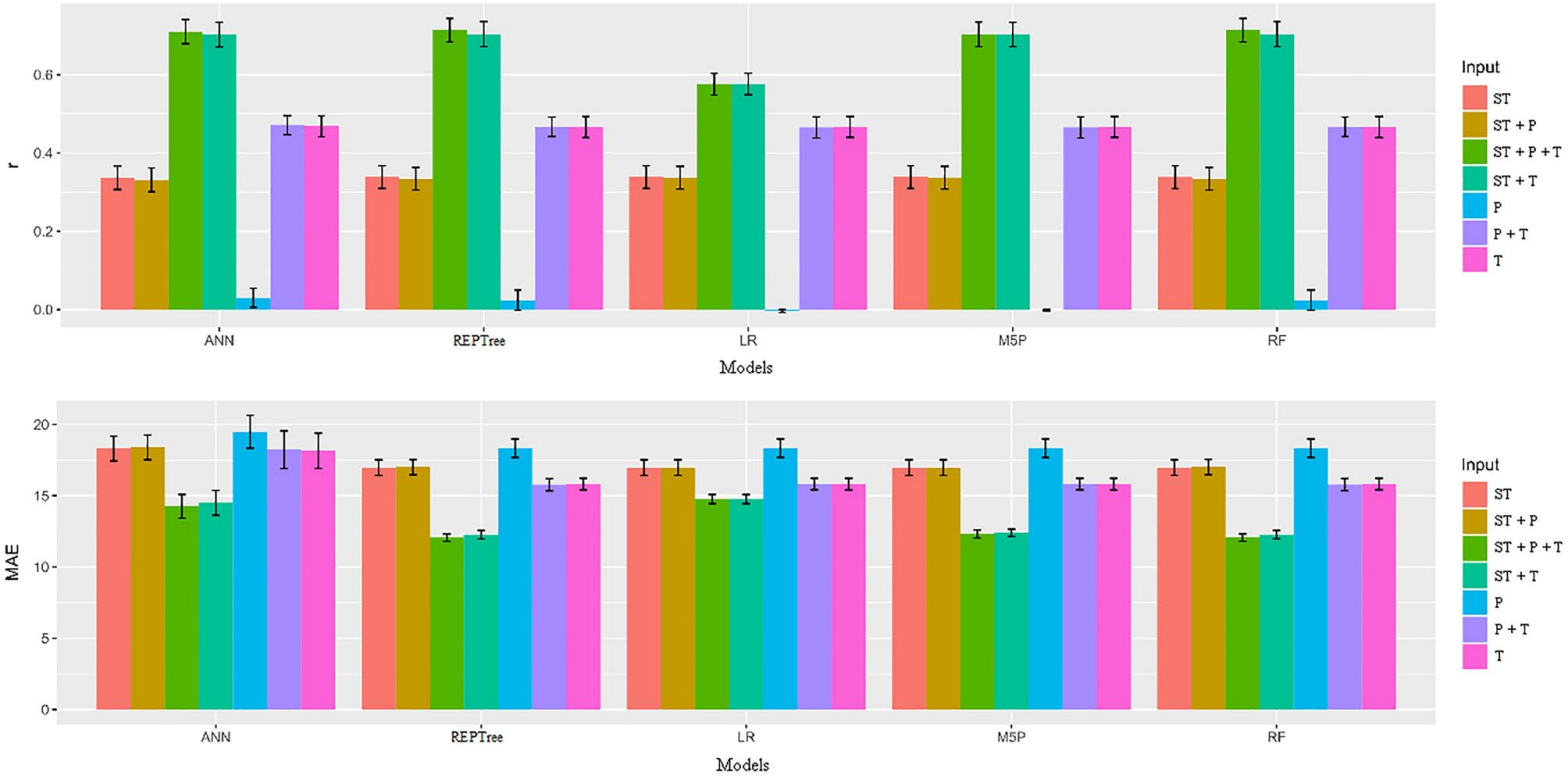
Mean values and scatter plot for the variables Pearson's correlation coefficient (r) and mean absolute error (MAE) between observed and estimated values of vigor in soybean seeds by different machine learning models and inputs.

The results shown in Fig. 6 indicate that the REPTree, M5P and RF models, when associated with the inputs T + P + ST, T + ST, P + ST and ST provided the highest r values (0.68 to 0.47) and the lowest MAE values. Ferreira et al^9^ found that seed storage at low temperatures (T + ST) reduced metabolic activity and maintained physiological quality. However, the choice of the combinations T + P + ST, T + ST was justified when analyzing the values of the mean absolute errors. The MAE for T + P + ST was 13.09, and for T + ST the values were 13.24. Importantly, although the ANN obtained good r results with the aforementioned inputs, the model showed high MAE values for all inputs compared to the LR model.

Seed vigor was mainly influenced by temperature and storage time, as was the case for the other variables evaluated. RF was the model that best predicted the vigor indices of the seeds using a smaller amount of data. The superior performance of RF possibly occurred due to the internal structure of the algorithm, which is based on multiple decision tree sets.

RF regression has advantages when predictor or explanatory variables are highly correlated, which is especially true for the variables temperature and storage time evaluated here. Variable collinearity can be a critical problem in traditional prediction models that are derived from linear regression^21,42,43^. Moreover, RF has been considered superior to other machine learning algorithms because it can easily handle many model parameters, reduce estimate bias, and has no problems with overfitting^18^. Recent studies have classified RF as an effective and versatile machine learning method for crop yield predictions^19^. To date, there are no studies for predicting storage seed quality from conditioning variables using ML models. Our study shows that it is possible to obtain satisfactory accuracy in predicting quality variables of stored soybean seeds using computational intelligence techniques, especially by employing the RF model. Furthermore, our findings provide support for decision-making about which conditioning variables should be evaluated and included in such prediction models, contributing to a more efficient soybean seed processing.

## Conclusion

The preservation of seed quality involves controlling the storage environment and the use of technology, such as packaging, that allow reducing the metabolic activity of the seeds over time. In this study, evaluating the predicting the quality of soybean seeds stored in different environments and packaging using Machine Learning, it was concluded that:

- The combination of input variables temperature and storage time was the best predictor of soybean seed quality indices during the storage period. The input variable packaging did not influence predicting the physiological quality of soybean. The packaging effect was suppressed by the low storage temperatures, allowing the same results to be achieved, but using a smaller number of input variables.
- The ML techniques outperformed the proposed control model (linear regression). Random Forest algorithm was the one that best predicted the physiological quality indices of the seeds during the storage period with a smaller amount of data, making it possible to better conduct overfitting problems. On the other hand, the Artificial Neural Network had the highest errors (MAE).

The proposed approach stood out in terms of speed compared to the analysis methods routinely used, making the processes more robust and with low operational costs compared to the laboratory analysis strategies traditionally used. Using ML can be an auxiliary tool for decision-making within the seed storage environment, thereby contributing to loss reduction.

## Data Availability

All research data and materials are available in the article.
